# Case Report of Spontaneous Bilateral Ovarian Pregnancy in a Nulliparous Lady

**DOI:** 10.1155/2021/6670763

**Published:** 2021-05-14

**Authors:** Abdulrahman D. Mahroofi, Jawaher K. Alsaqer, Nawal Saad Alabdulla, Rihab Ismael, Stephanie Hsu, M. Samy Ismail

**Affiliations:** ^1^King Hamad University Hospital, Al Sayh, Bahrain; ^2^Department of Obstetrics and Gynaecology, King Hamad University Hospital, Al Sayh, Bahrain; ^3^Research Department, King Hamad University Hospital, Al Sayh, Bahrain

## Abstract

Nontubal ectopic pregnancies, especially ovarian ones, are rare. Here, we report a case of spontaneous bilateral ovarian pregnancy in a 23-year-old nulliparous lady who presented with a three-day history of abdominal pain localized to the right iliac fossa. Laboratory investigations and pelvic US and transvaginal US findings were suggestive of a right ovarian ectopic pregnancy and left ovarian cyst. Following the patient's consent, the gynaecologist laparoscopically removed the right ovarian ectopic pregnancy and performed a left ovarian cystectomy. Histopathology revealed findings of trophoblastic tissue and chorionic villi with products of conception in both ovaries leading to the diagnosis of bilateral spontaneous ectopic pregnancy. Physicians must be mindful in cases that have a similar clinical presentation because an early diagnosis leads to a reduction in the morbidity and mortality of this specific patient population and helps to improve their overall prognosis.

## 1. Introduction

One of the most common causes of maternal mortality in the first trimester is ectopic pregnancy [1]. It is defined as implantation of the trophoblast outside the endometrium of the uterine cavity with an estimated incidence of 1.5% to 2% in all pregnancies [2]. The vast majority of these ectopic pregnancies happen in the fallopian tubes with the remaining being nontubal in origin [1].

Although an ovarian pregnancy is the most common type of nontubal ectopic pregnancy [3], it has an incidence of only 1/7000-1/40,000 live births and 0.5-3% of all ectopic gestations [4].

Primary bilateral ectopic pregnancy is a condition in which there are at least two concomitant spontaneous pregnancies in the same patient, with both being located in structures of the opposite side [2].

An increasing number of simultaneous bilateral ectopic pregnancies have been reported since the first case that was published by Bledsoe in 1918, of which a significant number have been the result of assisted reproductive techniques (ART). These techniques encompass a multitude of approaches such as ovulation induction, intrauterine insemination, in vitro fertilization, embryo transfer (IVF-ET), and intracytoplasmic sperm injections (ICSI) [2].

Frequent use of ART due to infertility seems to be the main factor responsible for accelerating the incidence rate of ovarian pregnancy to 6% among all ectopic pregnancies. Bilateral ovarian pregnancy has been reported in a case involving a patient who had received IVF-ET treatment for secondary infertility and in another case where a young patient had previously undergone intrauterine insemination (IUI) and controlled ovarian stimulation (COS) [4, 5].

Unlike tubal pregnancies which hold a 15% chance of recurrence, there have been no case reports of a repeat ovarian pregnancy to the best of our knowledge which indicates that a previous ovarian pregnancy may not be a risk factor for its recurrence [6].

The most frequent clinical symptom of an ovarian pregnancy is chronic pelvic pain. As this is a vague symptom, a high index of clinical suspicion is needed. The site of pain is also not specific as it can be felt in other sites, leading to the frequent misdiagnosis of a ruptured corpus luteal cyst in 75% of these cases. An adnexal mass can sometimes be palpable on examination [3].

A preoperative diagnosis of ovarian pregnancy is neither easy nor straightforward. Some criteria combine biochemical and ultrasonography findings which can include the following: a serum *β*hCG level ≥ 1000 IU/L, absence of a uterine gestational sac on transvaginal ultrasound, surgical confirmation of ovarian involvement, association with bleeding, visualization of chorionic villi, presence of an atypical cyst on the ovary, intact tubes, and negative serum *β*hCG after treatment [7].

The diagnosis of ovarian pregnancy is mostly made during surgery after histological confirmation. Even then, a correct diagnosis during surgery is only possible in 28% of cases due to difficulty in differentiating an ovarian pregnancy from haemorrhagic corpus luteum intraoperatively [3]. An anatomical method used to aid in the diagnosis is known as the Spielberg criteria, which depends on the following histopathological findings: the gestational sac is located in the region of the ovary, the ectopic pregnancy is attached to the uterus by the ovarian ligament, presence of ovarian tissue in the wall of the gestational sac is proven histologically, and the tube on the involved side is intact [4].

This case report highlights the importance of having an ovarian pregnancy as a differential diagnosis because of its clinical urgency and associated high maternal morbidity and mortality.

## 2. Case Report

A 23-year-old Bahraini female presented to the emergency department with a three-day history of generalized abdominal pain localizing to the right iliac fossa. The pain started gradually then increased in severity, radiating to her right shoulder. These symptoms were associated with nausea, five episodes of loose motion over the course of one day but no vomiting. The patient had no previous medical or surgical history and no known allergies. Her last menstrual period was on 16/07/2020, but on 14/08/2020, she had experienced vaginal bleeding for two days with blood clots similar in description to her menstrual period. Her expected date of delivery was documented as 22/04/2021; therefore, on presentation she was estimated to be at 6 weeks of gestation age.

Upon examination:

- *General look*: patient was conscious; alert; oriented to time, place, and person; vitally stable; and afebrile
- *Abdominal examination*: a soft, lax abdomen with tenderness over the suprapubic region and right iliac fossa. Her pain score was graded as five out of ten on the pain scale
- *Reflexes*: positive rebound tenderness was elicited, and rigidity of the abdomen was noticed
- Vaginal examination was not done

**Figure 1:**
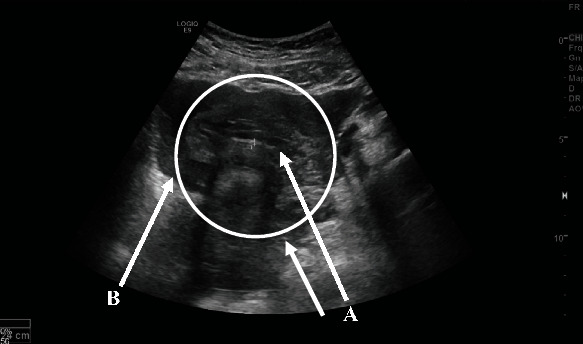
Pelvic sonography showing an empty uterus with thin endometrium (A) and free fluid (B).

**Figure 2:**
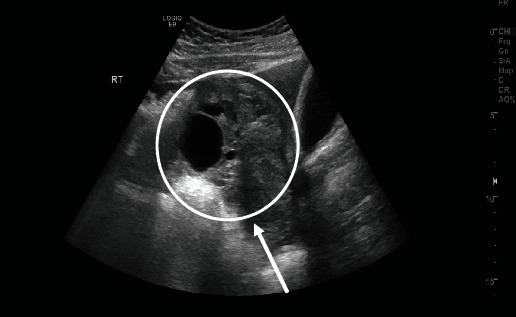
Pelvic sonography of the right ovary, visualizing an ectopic pregnancy.

Laboratory investigations revealed a *β*hCG level of 8442.3 mIU/mL and a positive urine pregnancy test. An ultrasound of the pelvis was performed by a radiologist, which revealed that the uterus was bulky, measuring 6 × 3 cm with an endometrial thickness of 9 mm and no identifiable intrauterine gestational sac (Figure 1). A right adnexal heterogeneous hypodense lesion was seen measuring 9.5 × 7 cm, most likely resembling an ectopic adnexal pregnancy (Figure 2). The right ovary was seen measuring 2.5 × 1.6 cm containing multiple cysts with the largest measuring 3.8 × 3.3 cm with normal vasculature on color Doppler. The left ovary was not seen. Pelvic free fluid was present bilaterally and estimated to be mild to moderate in amount. A bedside transvaginal ultrasound was then performed by a gynaecologist, which confirmed the patient's pelvic US findings and further revealed a left ovarian cyst measuring 3∗3 cm. In summary, the sonographic findings were highly suggestive of a right adnexal ectopic pregnancy and left ovarian cyst associated with mild to moderate free fluid.

After explaining to the patient and her husband regarding the findings of her condition and the need for surgical intervention, the couple agreed to a laparoscopic procedure under general anaesthesia with the potential to convert to a laparotomy with salpingectomy or oophorectomy if indicated. The patient was then transferred immediately to the operating theatre.

**Figure 3:**
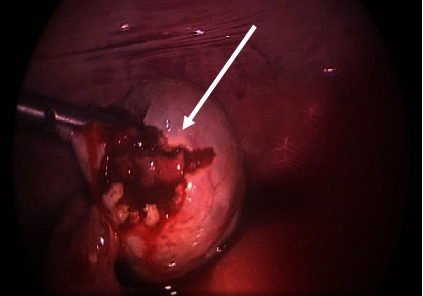
Laparoscopic view of the right ovary showing an ectopic pregnancy.

Laparoscopic findings confirmed distention of the right ovary with an ectopic pregnancy and a left ovarian cyst (Figure 3). The right ovarian ectopic was removed, and a left cystectomy was performed with preservation of the patient's ovaries and tubes. The removed tissue samples were then sent for analysis by histopathology. The total estimated blood loss of the surgery was 300 mL.

**Figure 4:**
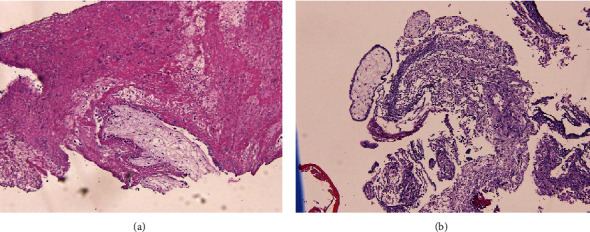
Histopathological view of the left ovarian tissue showing trophoblastic tissue (a) and chorionic villi (b).

**Figure 5:**
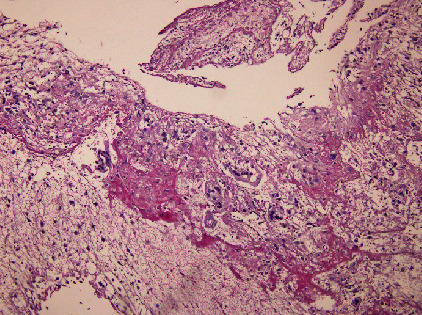
Histopathological view of the right ovarian tissue showing the chorionic villi.

Histopathological reports of both ovaries revealed the presence of ovarian parenchyma and fragments of cysts lined by luteinized cells with a focal area showing trophoblastic tissue and chorionic villi. Products of conception were seen in both the right and left ovaries, thus making the diagnosis consistent with a bilateral ovarian ectopic pregnancy (Figures 4 and 5).

The patient was discharged in stable condition and followed up regularly for postoperative care.

## 3. Discussion

A good prognosis of primary ovarian ectopic pregnancy necessitates early diagnosis and immediate intervention. This helps to decrease the incidence of maternal mortality and morbidity and preserve future fertility, as what was done in our case. Its diagnosis can be challenging among other types of nontubal pregnancies [8]. According to a reported case in 2017, misinterpretation of ultrasonographic findings remains the leading cause of missed early diagnosis [6]. However, with improvement of sonogram sensitivity and the widespread applications of ultrasound, earlier identification of the location of the gestational sac has become possible and guides the gynaecologist need to choose the appropriate management for ectopic pregnancies [9].

In the current case, we suspected a unilateral ovarian ectopic pregnancy preoperatively based on the patient's positive urine pregnancy test, high *β*hCG level, and ultrasonography findings. Intraoperatively, we found that the right ovary was distended with an ectopic pregnancy and left ovarian cyst. Both were excised, and tissue samples were sent for analysis by the histopathology department. Trophoblastic tissue, chorionic villi, and products of conception were present in both ovarian parenchyma; thus, bilateral ovarian ectopic pregnancy was surprisingly confirmed.

A majority of previous case reports have agreed on the rarity of a primary ovarian ectopic pregnancy and on the difficulty of its presentation as well as diagnosis preoperatively [1, 5, 6, 8]. Management of primary ovarian ectopic pregnancies can differ between cases. Options include being managed by laparotomy [8], medical treatment, or laparoscopy, which aligned with our case [10].

## 4. Conclusion

A spontaneous bilateral ovarian pregnancy is rare. It may present without the classical symptoms of ectopic pregnancy and haemorrhage, which is a life-threatening diagnosis if missed and thus considered to be a leading cause of maternal mortality in the first trimester accounting from 4% to 10% of all pregnancy-related deaths [11]. When there is suspicion of an ectopic pregnancy, ovarian locations should be kept in mind as early detection helps to determine timely intervention and management for these patients, which in turn improves the overall prognosis [12]. Hence, the clinical perception of gynaecologists and radiologists is crucial. No correlation has yet been established between a history of infertility or recurrent extrauterine pregnancy with ovarian pregnancy [11]. The presented case is an example of bilateral ovarian ectopic pregnancy in a nulliparous young lady, whose diagnosis was only confirmed after the histopathology report.

## Data Availability

The datasets analyzed are available from the corresponding author upon reasonable request.
